# Supplementary material for: CinA mediates multidrug tolerance in Mycobacterium tuberculosis
Source: Nat Commun. 2022 Apr 22;13:2203. doi: 10.1038/s41467-022-29832-1 (PMC9033802; doi:10.1038/s41467-022-29832-1)
Supplement: Supplementary file 1 — Supplementary Information [file 41467_2022_29832_MOESM1_ESM.pdf]

Supplementary Information

CinA mediates multidrug tolerance in *Mycobacterium tuberculosis*

Kaj Kreutzfeldt, Robert S. Jansen, Travis Hartman, Alexandre Gouzy, Ruojun Wang, Inna Krieger, Matthew D. Zimmerman, Martin Gengenbacher, Jansy Sarathy, Min Xie, Véronique Dartois, James C. Sacchettini, Kyu Y. Rhee, Dirk Schnappinger, Sabine Ehrt

Supplementary Figures 1 - 6

Supplementary Table 1

Supplementary Methods

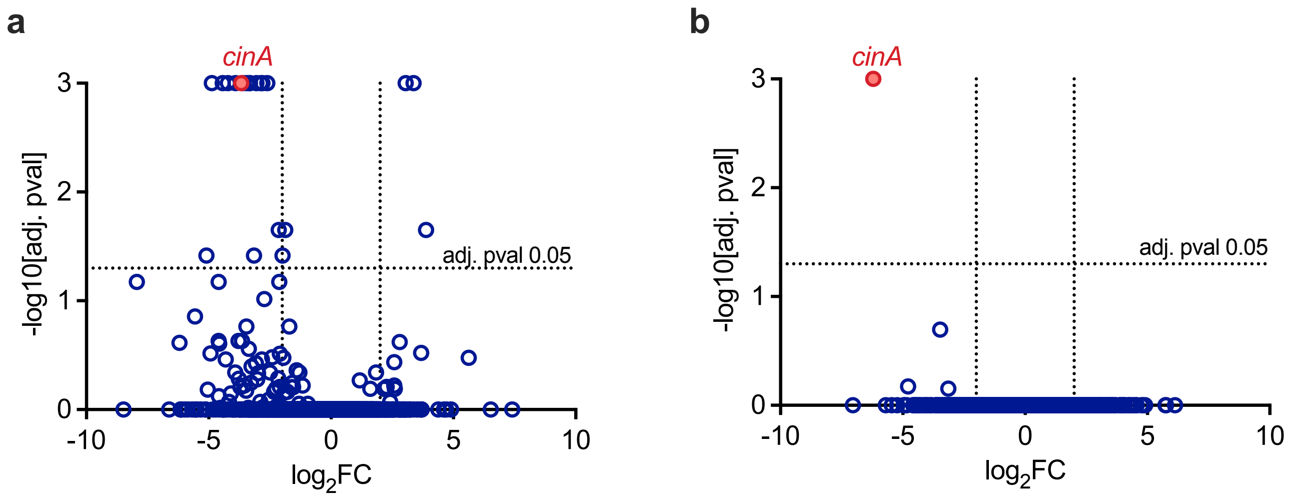

Supplementary Fig. 1. TnSeq identifies *cinA* to mediate tolerance to isoniazid in macrophages and in vitro. Volcano plots of TnSeq data depicting relative mutant abundance after (**a**) two 120 h cycles of residence in IFN-γ activated and isoniazid-treated primary bone marrow-derived macrophages compared to abundance before treatment with isoniazid and (**b**) culture in PBS for 28 days with isoniazid treatment during the last 14 days compared to the input culture. Stippled vertical lines indicate a $\log_2$ fold change of +/- 2 and stippled horizontal lines indicate an adjusted *P* value of 0.05. To determine statistical significance a nonparametric permutation test was used to calculate P values. Q values (adj. val) were obtained by adjusting for multiple comparisons using the Benjamin-Hochberg correction. Genes with a Q value of <0.05 according to the permutation test were considered to be significant determinants of fitness in the presence of INH.

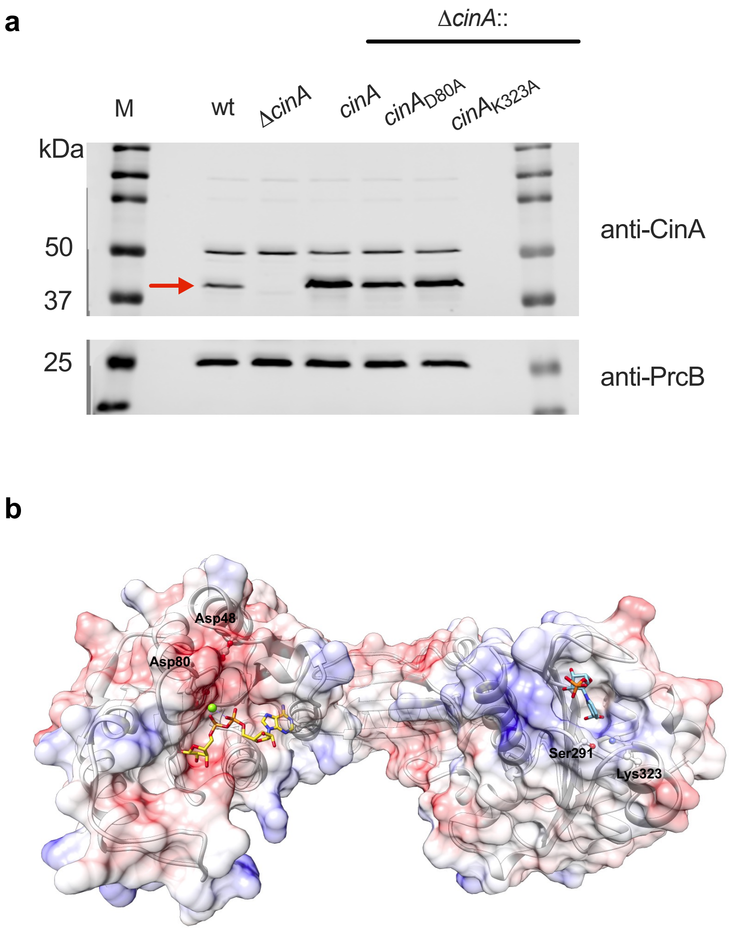

Supplementary Fig. 2. Validation of the *Mtb*Δ*cinA* mutant, expression of mutated CinA proteins and a CinA structure homology model. a Immunoblotting confirming loss of CinA in Δ*cinA* and expression of CinA and mutated CinA proteins. Whole cell lysates were prepared from wild type (wt), Δ*cinA*, Δ*cinA* ::*cinA*, Δ*cinA* ::*cinA*$_{D80A}$ *and* Δ*cinA* ::*cinA*$_{K323A}$ mid-log-phase cultures. Protein (40µg) was separated using SDS-PAGE and CinA protein (marked by red arrow) was detected using rabbit anti-CinA sera. PrcB was used as loading control. The experiment was performed three times with similar results. **b** Homology model of the CinA structure created based on the structure of the CinA enzyme from *Thermus thermophilus.* Indicated are the positions of the two active-site aspartate residues of the pyrophosphatase domain (left) with di-phosphoribose in its binding pocket and the Mg^{2+} ion (green sphere) that is required for activity (from the superimposed 4UUX structure; https://www.rcsb.org/structure/4UUX). The nicotinamide mononucleotide deamidase domain is shown on the right with the serine and lysin residues of active site positions and the nicotinate mononucleotide enzymatic product bound (from superimposed 4UOC structure; https://www.rcsb.org/structure/4UOC).

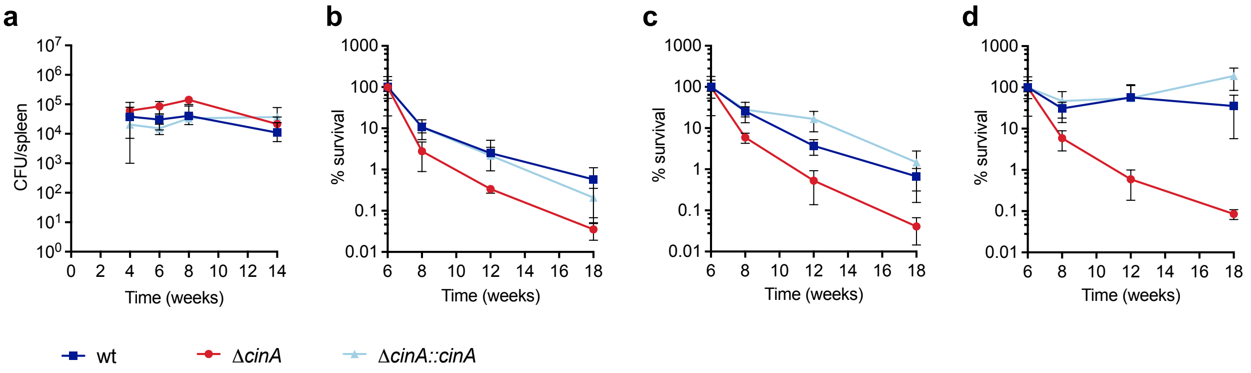

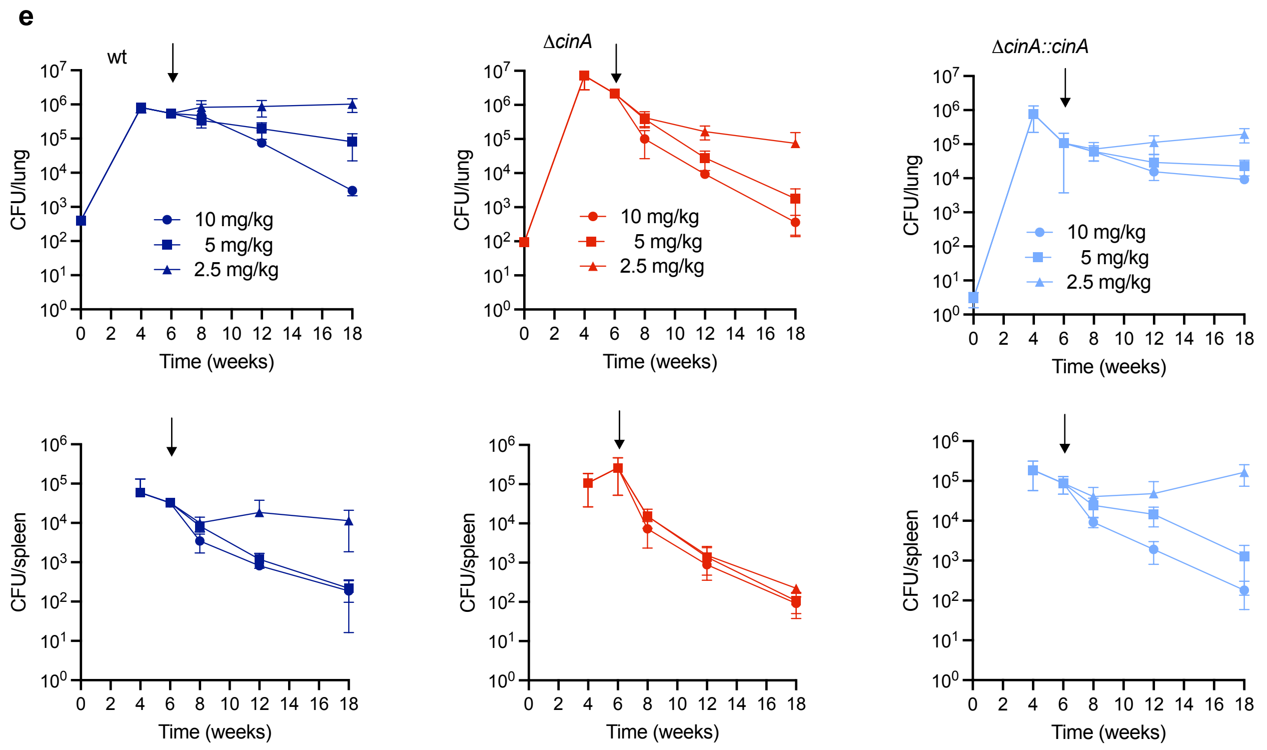

Supplementary Fig. 3. Deletion of *cinA* potentiates isoniazid activity during chronic mouse infection. **a** Bacterial titers from spleens of C57BL/6 mice infected with the indicated strains. **b-d** C57BL/6 mice were infected with the indicated strains and treated with isoniazid (**b**) 10 mg/kg/d (**c**) 5 mg/kg/d (**d**) 2.5 mg/kg/d starting after six weeks of infection. CFU are from spleen homogenates and normalized to the beginning of isoniazid treatment. Mice received isoniazid in drinking water ad libitum. Data are means ± SD of four mice (except in d week six, wt n=3) and are representative of two independent infections for wt and Δ*cinA*. In the second experiment drug treatment started after four weeks of infection with similar impact on bacterial survival as observed here. **e** CFU quantifications of the experiment shown in Fig. **2 b-d** and in **b-d**. The arrows indicate start of treatment with isoniazid. Data are means ± SD of three to four mice and are representative of two independent infections for wt and Δ*cinA*.

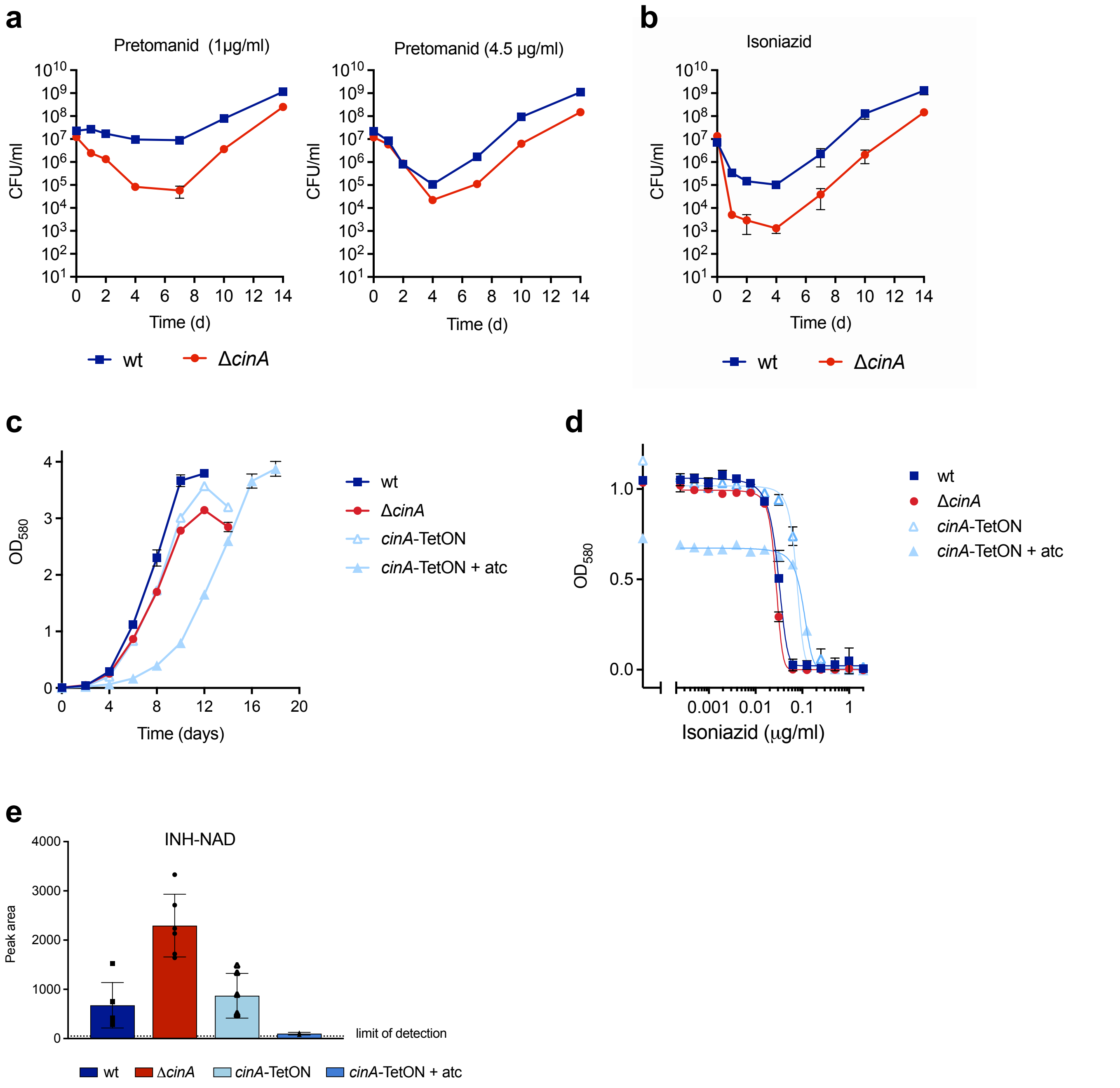

Supplementary Fig. 4. Development of drug resistance and impact of CinA overexpression on growth and the MIC of isoniazid. a Prolonged kill curves with pretomanid concentrations that are ~ 15 x MIC against Δ*cinA* (1 µg/ml) and 15 x the MIC against wile type *Mtb* (4.5 µg/ml). **b** Prolonged kill curves with isoniazid (0.5 µg/ml). **c** Growth curves of the indicated strains. **d** Impact of isoniazid on growth of the indicated strains. Data are means ± SD of triplicate cultures and representative of two experiments. **e** Quantification of the INH-NAD adduct in the indicated strains. Data are means ± SD of six independent cultures. In the case of *cinA*-TetON + atc the INH-NAD adduct was below the limit of detection in four out of the six samples and below the limit of quantitation in the remaining two.

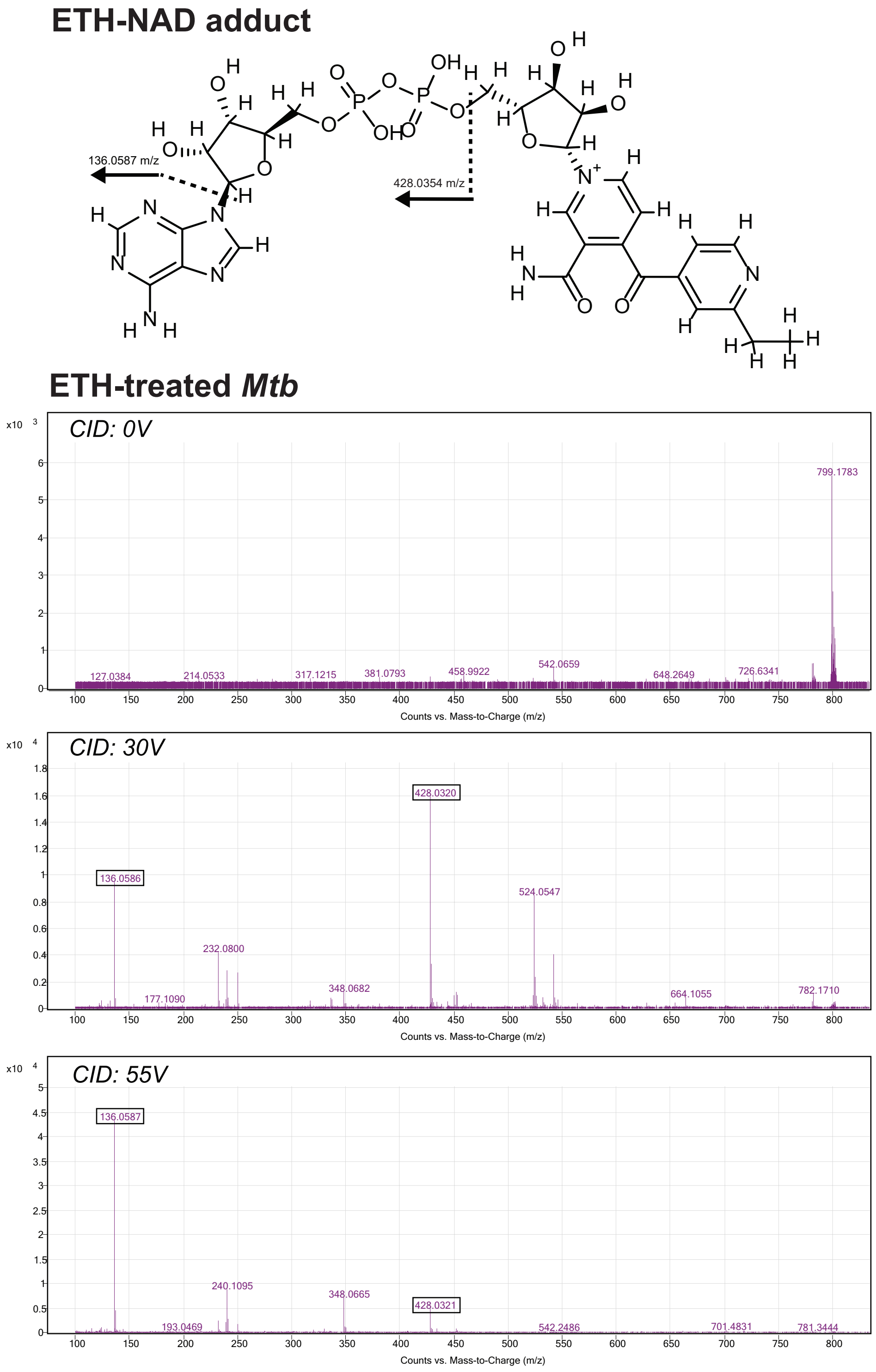

Supplementary Fig. 5. LC-MS/MS chromatogram of ethionamide (ETH)-treated *Mtb*. MS/MS fragmentation spectra of a mass ion corresponding to the predicted mass of an ETH-NAD adduct observed in ethionamide-treated *Mtb*. The annotated masses provide confirmatory evidence of mass matching to the predicted parent adduct (Δppm = 10) and fragments corresponding to the adenine and ADP moieties of the ETH-NAD adduct.

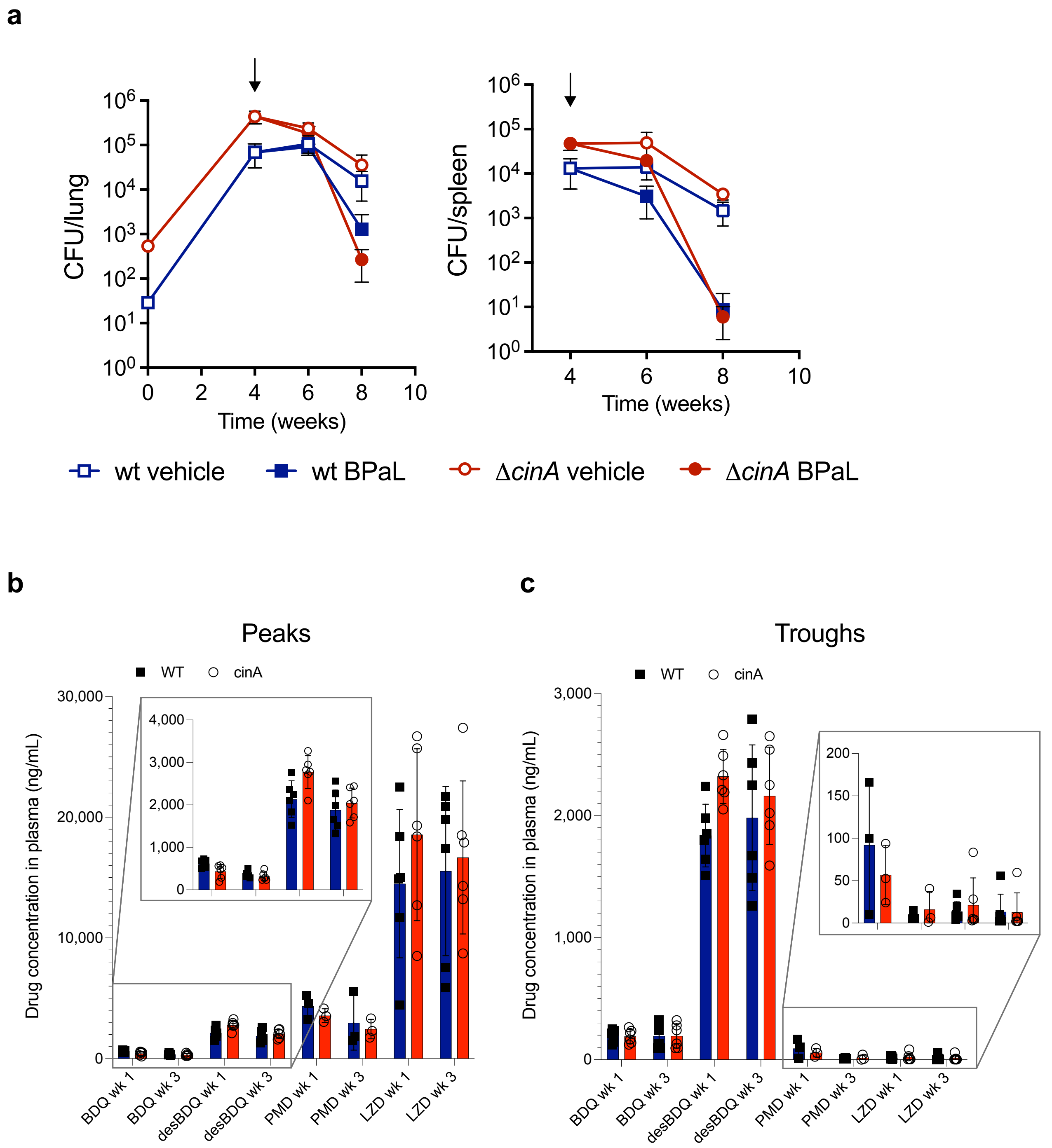

Supplementary Fig. 6. Deletion of *cinA* potentiates the efficacy of BPaL. A CFU quantifications of the experiment shown in **Fig. 6**. The arrows indicate start of treatment with BPaL. Means and standard deviations are shown for groups of three to six mice. **b,c** Therapeutic drug monitoring in mice on treatment with bedaquiline (BDQ), pretomanid (PMD) and linezolid (LZD)**. b** Peak and (**c)** trough plasma concentrations of bedaquiline, its major metabolite desmethyl-BDQ, pretomanid and linezolid collected in three to six mice per group after one and three weeks of daily treatment, as indicated. Data were obtained on the same days from mice infected with wild type H37Rv and Δ*cinA* mutant to ensure similar drug exposure in the two mouse cohorts. Data are means ± SD of three to six samples. All drug levels were consistent across animals within a group, on both occasions (week one and three) and across mice infected with wild type H37Rv and Δ*cinA*.

Supplementary Table 1. Drug susceptibility of wt *Mtb*, Δ*cinA* and *cinA*-TetON.

Compound	wt		Δ*cinA*		*cinA*-TetON	
	MIC_{50}	MIC	MIC_{50}	MIC	MIC_{50}	MIC
**Isoniazid**	0.030 - 0.036	0.051 - 0.055	0.020 - 0.026	0.029 - 0.038	0.069 - 0.076	0.112 - 0.115
**Ethionamide**	0.165 – 0.356	0.218 - 0.552	0.085 - 0.180	0.135 - 0.270	0.420 - 0.682	0.695 - 1.169
**Delamanid**	0.0141 - 0.316	0.027 -0.089	0.008 - 0.025	0.015 - 0.078	0.010 - 0.057	0.019 - 0.149
**Pretomanid**	0.153 - 0.185	0.219 - 0.315	0.039	0.041 - 0.043	0.175 - 0.192	0.275 - 0.305
**Rifampicin**	0.008 - 0.018	0.012 - 0.033	0.008 - 0.017	0.015 - 0.036	0.006 - 0.015	0.010 - 0.022

MIC values are calculated from two or three independent experiments each with triplicate cultures. Bacteria were grown in 7H9 medium supplemented with 0.2% glycerol, 0.05% Tween-80, and ADN (0.5% bovine serum albumin, 0.2% dextrose, 0.085% NaCl).

MIC_{50}: Concentration (µg/ml) inhibiting bacterial growth to 50% of maximal growth. Values were determined using log(inhibitor) vs. response variable slope (four parameter) nonlinear regression analysis in the GraphPad Prism 8.4.2 software.

MIC: Lowest concentration (µg/ml) that completely inhibits bacterial growth. Values were determined by fitting the log(inhibitor) vs. response data to a Gompertz model using the template provided by GraphPad.

Supplementary Methods

Drug quantitation by high pressure liquid chromatography coupled to tandem mass spectrometry (HPLC-MS/MS)

HPLC-MS/MS analysis was performed on a Sciex Applied Biosystems Qtrap 6500+ triple-quadrupole mass spectrometer coupled to a Shimadzu Nexera X2 UHPLC system to quantify each drug in plasma. Neat 1 mg/ml DMSO stocks of BDQ, desmethyl BDQ, PMD, and LZD were serial diluted in 50/50 acetonitrile/water to create standard curves and quality control (QC) spiking solutions. Standards and QCs were created by adding 10 μl of spiking solutions to 90 μl of drug free plasma (CD-1 K2EDTA Mouse, Bioreclamation IVT). Twenty μl of control, standard, QC, or study sample were added to 200 μl of acetonitrile/methanol 50/50 protein precipitation solvent containing deuterated analogs as internal standards (10 ng/ml BDQ-d6, 100 ng/ml LZD-d8, and 20 ng/ml PMD-d5). Extracts were vortexed for 5 min and collected by centrifugation at 4000 RPM for 5 min. 100 μl of supernatant was transferred for HPLC-MS/MS analysis and diluted with 100 μl of Milli-Q deionized water. BDQ standard was received from Chemshuttle; desmethyl BDQ, LZD-d8, and BDQ-d6 were purchased from Clearsynth. LZD was purchased from Sigma-Aldrich. PMD was synthesized at Bioduro.

Chromatography was performed on an Agilent Zorbax SB-C8 column (2.1x30 mm; particle size, 3.5 μm) using a reverse phase gradient. Milli-Q deionized water with 0.1% formic acid was used for the aqueous mobile phase and 0.1% formic acid in acetonitrile for the organic mobile phase. Multiple-reaction monitoring (MRM) of parent/daughter transitions in electrospray positive-ionization mode was used to quantify the analytes. The following MRM transitions were used: BDQ 555.20/58.20, desmethyl-BDQ 541.30/480.30, PMD 360.00/175.00, LZD 338.00/235.00, BDQ-d6 561.26/64.20, PMD-d5 365.0/175.0, and LZD-d8 346.15/304.20. Sample analysis was accepted if the concentrations of the quality control samples were within 20% of the nominal concentration. Data processing was performed using Analyst software (version 1.6.2; Applied Biosystems Sciex).
