# Supplementary material for: CinA mediates multidrug tolerance in Mycobacterium tuberculosis
Source: Nat Commun. 2022 Apr 22;13:2203. doi: 10.1038/s41467-022-29832-1 (PMC9033802; doi:10.1038/s41467-022-29832-1)
Supplement: Supplementary file 3 — Description of Additional Supplementary Files [file 41467_2022_29832_MOESM3_ESM.docx]

File name: Supplementary Data 1

Description: Whole-genome phenotypic profiling of *M. tuberculosis* mutants in IFNγ-activated, isoniazid-treated bone marrow-derived macrophages and during PBS starvation in the presence of isoniazid.
