# Supplementary material for: CinA mediates multidrug tolerance in Mycobacterium tuberculosis
Source: Nat Commun. 2022 Apr 22;13:2203. doi: 10.1038/s41467-022-29832-1 (PMC9033802; doi:10.1038/s41467-022-29832-1)
Supplement: Supplementary file 5 — Reporting Summary [file 41467_2022_29832_MOESM5_ESM.pdf]

nature research

Corresponding author(s): Sabine Ehrt

Last updated by author(s): 2022/03/24

Reporting Summary

Nature Research wishes to improve the reproducibility of the work that we publish. This form provides structure for consistency and transparency in reporting. For further information on Nature Research policies, see our Editorial Policies and the Editorial Policy Checklist.

Statistics

For all statistical analyses, confirm that the following items are present in the figure legend, table legend, main text, or Methods section.

n/a	Confirmed	
☐	☒	The exact sample size (*n*) for each experimental group/condition, given as a discrete number and unit of measurement
☐	☒	A statement on whether measurements were taken from distinct samples or whether the same sample was measured repeatedly
☐	☒	The statistical test(s) used AND whether they are one- or two-sided<br>*Only common tests should be described solely by name; describe more complex techniques in the Methods section.*
☒	☐	A description of all covariates tested
☐	☒	A description of any assumptions or corrections, such as tests of normality and adjustment for multiple comparisons
☐	☒	A full description of the statistical parameters including central tendency (e.g. means) or other basic estimates (e.g. regression coefficient) AND variation (e.g. standard deviation) or associated estimates of uncertainty (e.g. confidence intervals)
☐	☒	For null hypothesis testing, the test statistic (e.g. *F*, *t*, *r*) with confidence intervals, effect sizes, degrees of freedom and *P* value noted<br>*Give P values as exact values whenever suitable.*
☒	☐	For Bayesian analysis, information on the choice of priors and Markov chain Monte Carlo settings
☒	☐	For hierarchical and complex designs, identification of the appropriate level for tests and full reporting of outcomes
☒	☐	Estimates of effect sizes (e.g. Cohen's *d*, Pearson's *r*), indicating how they were calculated

Our web collection on statistics for biologists contains articles on many of the points above.

Software and code

Policy information about availability of computer code

Data collection	Prism (version 9.3.1, GraphPad)
Data analysis	Prism (version 9.3.1, GraphPad); Analyst software (version 1.6.2; Applied Biosystems Sciex);TRANSIT Version 3.2.3 TnSeq analysis software tool which includes the pre-processing tool TPP.

For manuscripts utilizing custom algorithms or software that are central to the research but not yet described in published literature, software must be made available to editors and reviewers. We strongly encourage code deposition in a community repository (e.g. GitHub). See the Nature Research guidelines for submitting code & software for further information.

Data

Policy information about availability of data

All manuscripts must include a data availability statement. This statement should provide the following information, where applicable:

- Accession codes, unique identifiers, or web links for publicly available datasets
- A list of figures that have associated raw data
- A description of any restrictions on data availability

Data generated during the study are available within the paper and/or its supplement. Source data are provided with this paper. We used the Mtb H37Rv reference genome (GenBank accession number NC_018143.1: https://www.ncbi.nlm.nih.gov/nuccore/NC_018143) to map transposon-chromosome-junctions.

Field-specific reporting

Please select the one below that is the best fit for your research. If you are not sure, read the appropriate sections before making your selection.

☒ Life sciences ☐ Behavioural & social sciences ☐ Ecological, evolutionary & environmental sciences

For a reference copy of the document with all sections, see nature.com/documents/nr-reporting-summary-flat.pdf

Life sciences study design

All studies must disclose on these points even when the disclosure is negative.

Sample size	Animal studies were powered according to historical variance across animals. Based on previous experiments with multiple Mtb mutants, we calculated that to detect a minimum difference in CFU of 1 log10 we will need 4-5 mice per group (a= 0.05, power = 95%) depending on organ and time point.
Data exclusions	No data were excluded from the analysis
Replication	Generally, we performed at least two biologically independent experiments with several replicates. Details are specified for each experiment in the manuscript, methods and/or figure legends.
Randomization	Mice were randomly chosen to be infected with the wild type, the mutant or the complemented mutant. For in vitro experiments, randomization was not relevant because there were no assigned groupings.
Blinding	Investigators were generally not blinded during data collection or analysis. All data collected were quantifiable and not subject to biases that might be introduced by the experimenter.

Reporting for specific materials, systems and methods

We require information from authors about some types of materials, experimental systems and methods used in many studies. Here, indicate whether each material, system or method listed is relevant to your study. If you are not sure if a list item applies to your research, read the appropriate section before selecting a response.

Materials & experimental systems

n/a	Involved in the study	
☐	☒	Antibodies
☒	☐	Eukaryotic cell lines
☒	☐	Palaeontology and archaeology
☐	☒	Animals and other organisms
☒	☐	Human research participants
☒	☐	Clinical data
☒	☐	Dual use research of concern

Methods

n/a	Involved in the study	
☒	☐	ChIP-seq
☒	☐	Flow cytometry
☒	☐	MRI-based neuroimaging

Antibodies

Antibodies used	A rabbit polyclonal antibody against recombinant, purified CinA was generated by Thermofisher Scientific and used in this study. Poly-clonal rabbit anti PckA and anti-PrcB antibodies were uses as controls.
Validation	The antibody bound to CinA from WT Mtb and to recombinant CinA. It did not detect any CinA protein in the CinA deletion mutant. Anti-PckA and anti-PrcB antibodies have been previously validated in our lab through the detection of recombinant proteins and lack of detection of the proteins in lysates of deletion mutants of these proteins.

Animals and other organisms

Policy information about studies involving animals; ARRIVE guidelines recommended for reporting animal research

Laboratory animals	Female, seven to eight week old mice (Mus musculus C57BL/6 from Jackson Labs) were used in this study. The mice were housed in in HEPA-filtered cages within the ABSL-3 facility with a 12hr light-dark cycle at 70F +/- 2F with 50% humidity (range is 30-70%).
Wild animals	The study did not involve wild animals
Field-collected samples	The study did not involve samples collected from the field
Ethics oversight	Mouse experiments were performed in accordance with the Guide for the Care and Use of Laboratory 302 Animals of the National

Institutes of Health, with approval from the Institutional Animal Care and Use Committee of Weill Cornell Medicine (protocol # 0601-441A)

Note that full information on the approval of the study protocol must also be provided in the manuscript.
